# New Mouse Lines for the Analysis of Neuronal Morphology Using CreER(T)/loxP-Directed Sparse Labeling

**DOI:** 10.1371/journal.pone.0007859

**Published:** 2009-11-16

**Authors:** Tudor C. Badea, Zhong L. Hua, Philip M. Smallwood, John Williams, Thomas Rotolo, Xin Ye, Jeremy Nathans

**Affiliations:** 1 Department of Molecular Biology and Genetics, Johns Hopkins University School of Medicine, Baltimore, Maryland, United States of America; 2 Department of Neuroscience, Johns Hopkins University School of Medicine, Baltimore, Maryland, United States of America; 3 Department of Ophthalmology, Johns Hopkins University School of Medicine, Baltimore, Maryland, United States of America; 4 Howard Hughes Medical Institute, Johns Hopkins University School of Medicine, Baltimore, Maryland, United States of America; University of Nebraska Medical Center, United States of America

## Abstract

**Background:**

Pharmacologic control of Cre-mediated recombination using tamoxifen-dependent activation of a Cre-estrogen receptor ligand binding domain fusion protein [CreER(T)] is widely used to modify and/or visualize cells in the mouse.

**Methods and Findings:**

We describe here two new mouse lines, constructed by gene targeting to the *Rosa26* locus to facilitate Cre-mediated cell modification. These lines should prove particularly useful in the context of sparse labeling experiments. The *R26rtTACreER* line provides ubiquitous expression of CreER under transcriptional control by the tetracycline reverse transactivator (rtTA); dual control by doxycycline and tamoxifen provides an extended dynamic range of Cre-mediated recombination activity. The *R26IAP* line provides high efficiency Cre-mediated activation of human placental alkaline phosphatase (hPLAP), complementing the widely used, but low efficiency, *Z/AP* line. By crossing with mouse lines that direct cell-type specific CreER expression, the *R26IAP* line has been used to produce atlases of labeled cholinergic and catecholaminergic neurons in the mouse brain. The *R26IAP* line has also been used to visualize the full morphologies of retinal dopaminergic amacrine cells, among the largest neurons in the mammalian retina.

**Conclusions:**

The two new mouse lines described here expand the repertoire of genetically engineered mice available for controlled *in vivo* recombination and cell labeling using the Cre-lox system.

## Introduction

Altering the mouse genome by Cre-mediated recombination is one of the central technologies of modern mammalian genetics [1]. An important adjunct to this technology has been the development of a method for pharmacologic control of Cre-mediated recombination by fusing Cre with a mutant estrogen receptor ligand-binding domain that recognizes 4-hydroxytamoxifen (4HT) rather than estrogen [CreER(T); referred to hereafter as CreER; 2,3]. The CreER protein is sequestered in the cytosol until 4HT exposure releases it for nuclear migration.

For typical applications of the CreER technology - for example, experiments involving timed inactivation of conditional alleles - highly efficient recombination is considered desirable. By contrast, relatively inefficient recombination is essential for experiments in which the goal is to monitor the behavior, morphology, or function of individual genetically modified cells in an environment populated by unmodified neighbors. In extreme cases - for example, experiments using genetically encoded reporters to study the morphologies of very large CNS neurons - the efficiency of recombination must be lowered to fewer than 20 cells per mouse [4]. Other applications have used sparse labeling with Cre/loxP recombination to study neuronal morphologies in the retina [5], to define the role of the Frizzled5 receptor in the survival of thalamic neurons [6], and to compare the roles of transcription factors Brn3a and Brn3b in retinal ganglion cell (RGC) development [7].

One limitation of the mouse lines currently available for sparse labeling relates to the efficiency of Cre-mediated recombination, which varies substantially with different combinations of CreER source and loxP target. For example, we observed that the *Z/AP* line, which expresses human placental alkaline phosphatase (AP) upon excision of a loxP-flanked beta-geo/transcriptional stop cassette [8], recombines with an efficiency ∼1,000 lower than similarly designed conditional AP knock-in alleles of the *Brn3a* and *Brn3b* genes (*Brn3a^CKOAP^* and *Brn3b^CKOAP^*), when each target gene is tested with a ubiquitously expressed *CreER* knock-in at the *Rosa26* locus (*R26CreER*; 7,9,10). In the absence of 4HT, *R26CreER;Z/AP* mice typically exhibit no AP+ cells per retina, whereas *R26CreER;Brn3a^CKOAP/+^* and *R26CreER;Brn3b^CKOAP/+^* mice exhibit several hundred AP+ RGCs per retina. A similar differential in the number of AP+ neurons is observed following in utero or early postnatal exposure to 4HT, with a single IP injection of 100–200 µg in the early postnatal period producing several dozen AP+ RGCs per *R26CreER;Z/AP* retina and many thousands of AP+ RGCs per *R26CreER;Brn3a^CKOAP/+^* or *R26CreER;Brn3a^CKOAP/+^* retina [7], [10].

For sparse labeling of neurons without selection for a particular subtype, the *R26CreER;Z/AP* combination with an early postnatal injection of 4HT generates a frequency of labeling that is close to optimal for morphologic analyses [(0.001%–0.01% of neurons labeled; 5,10]. When the *Z/AP* reporter is used in conjunction with a set of cell-type selective *CreER* lines – produced by knocking an *IRES-CreER* into the 3′ untranslated region (UTR) of the genes coding for choline acetyl transferase (*ChAT-IRES-CreER*) or tyrosine hydroxylase (*TH-IRES-CreER*) - the low level of CreER production, together with the relatively small number of catecholaminergic or cholinergic neurons, results in extremely sparse cell labeling [4].

In the present work we have enhanced the sparse labeling technique by developing two *Rosa26* knock-in mouse lines: one line increases the dynamic range of CreER action, and the second line exhibits Cre-mediated activation of AP with high efficiency and with undetectable background AP activity in unrecombined cells. The *R26rtTACreER* line uses the reverse tetracycline transactivator (rtTA) and doxycycline to control the expression of CreER, with the result that 4HT-induced Cre recombinase activity can be set at a level that is substantially lower than that typically obtained with constitutively expressed CreER lines. Thus the *R26rtTACreER* line permits precisely timed sparse recombination of target loci with a high efficiency of Cre-mediated recombination. Because the *Rosa26* locus is widely expressed, the *R26rtTACreER* line can be used to generate sparse genetic mosaics to test the behavior of individual mutant cells in a wide variety of tissues and/or in situations in which the conventional null allele is lethal. The *R26IAP* line complements the popular but inefficiently recombining *Z/AP* line by providing an AP reporter that exhibits high efficiency Cre-mediated recombination in virtually any cell. The *R26IAP* line should prove especially useful when paired with CreER loci that exhibit low levels of 4HT-induced Cre activity. We demonstrate the utility of the latter line by using it to: (a) assess the efficiency of Cre-mediated recombination following either direct uptake of Cre protein or infection by an adenovirus vector expressing Cre, (b) generate brain atlases of sparsely labeled cholinergic and catecholaminergic neurons, and (c) characterize the morphologies of type 1 dopaminergic retinal amacrine cells, which have axon-like arbors that are among the largest of any cells in the mammalian retina.

## Results

### *R26rtTACreER*: Dual Control of Cre Action by Doxycycline and 4-Hydroxytamoxifen (4HT)

**Figure 1:**
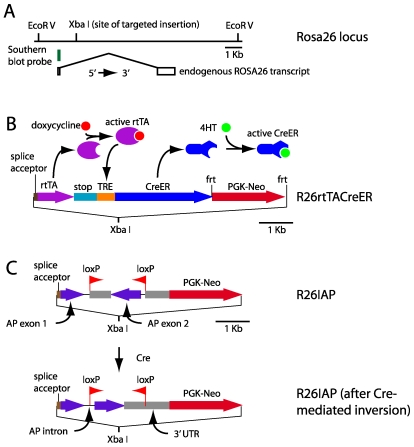
Structures of the *R26rtTACreER* and *R26IAP* knock-in alleles. (A) The WT *Rosa26* locus showing the location of the ubiquitous transcript derived from this locus and the unique *Xba* I site into which the *rtTACreER* and *IAP* cassettes were inserted. (B) The *R26rtTACreER* locus. The *rtTA* coding region is preceded by a splice acceptor so that it can be spliced onto the 5′ exon of the endogenous *Rosa26* transcript, and it is followed by multiple transcription termination signals (“stop”). In the presence of doxycycline, the rtTA protein activates transcription from the seven head-to-tail Tet response elements immediately 5′ of a minimal cytomegalovirus immediate early promoter (“TRE”), leading to expression of the CreER coding region. The resulting CreER protein translocates to the nucleus upon binding to 4-hydroxytamoxifen (4HT). (C) The *R26IAP* locus in its germline configuration (upper diagram) and following Cre-mediated recombination (lower diagram). The *IAP* region is preceded by a splice acceptor so that it can be spliced onto the 5′ exon of the endogenous *Rosa26* transcript. In the germline configuration, the two loxP sites in head-to-head orientation surround a central region that must be inverted to generate a correctly configured human placental alkaline phosphatase (*hPLAP*) minigene (lower diagram). In the orientation shown in the lower diagram, one loxP site resides in the intron and the second loxP site resides in the 3′ UTR. For the *R26rtTACreER* allele, the frt-flanked *PGK-Neo* selectable marker used during ES culture was removed by Flp-mediated recombination in the mouse germline. Scale bars in A, B and C are 1 Kb.

To construct a ubiquitously expressed *CreER(T)* allele (hereafter referred to as ‘*CreER*’; 2) with a large dynamic range of *CreER* expression, we generated the *Rosa26* knock-in allele *R26rtTACreER* (Figure 1A,B). In this allele, *CreER* is under the control of the reverse tetracycline trans-activator (rtTA), a sequence-specific DNA-binding protein that activates transcription upon binding tetracycline derivatives such as doxycycline [11]. The rtTA coding sequences are transcribed as part of the ubiquitously expressed Rosa26 transcription unit and are joined by splicing to the 5′ exon of this transcript. Upon administration of doxycycline (in food pellets and/or drinking water), rtTA activates transcription of the CreER coding region by binding to seven tandem copies of the Tet operator located immediately 5′ of a minimal promoter.

**Figure 2:**
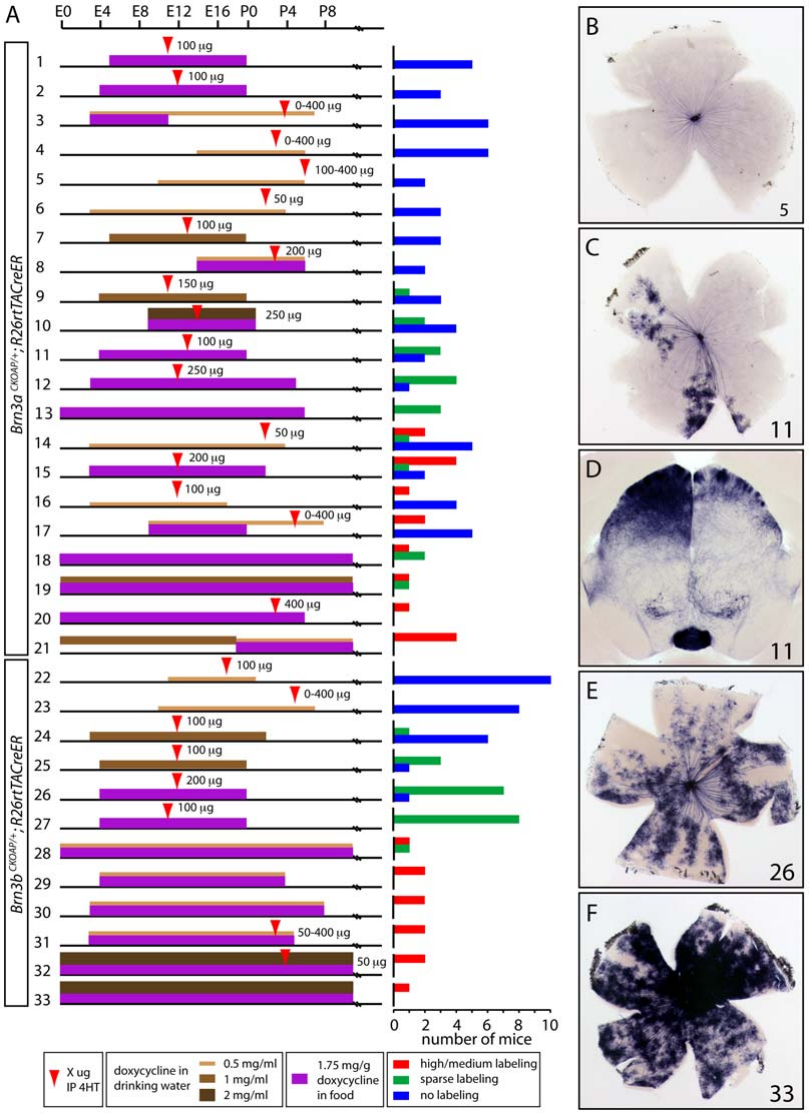
Dose and timing of doxycycline and 4HT exposure on Cre-mediated recombination efficiency using *R26rtTACreER*. (A) Individual experiments are numbered, the doxycycline and 4HT regimens are shown schematically, and the number of mice with high/medium, sparse, or no AP+ labeling of RGCs is shown in the color-coded histograms at right. Experiments 1–21 and 22–33 were performed with *R26rtTACreER;Brn3a^CKOAP/+^* and *R26rtTACreER;Brn3b^CKOAP/+^* mice, respectively. (B–F) Representative retina flat mounts (B,C,E,F) and a 200 µm coronal brain section at the level of the superior colliculus (D) were obtained from the numbered experiments indicated in the lower right corner of each panel. A single IP injection was used to deliver 4HT either to the mother for in utero exposure or to individual pups for postnatal exposure.

To systematically explore the relationship between the level and timing of doxycycline and 4HT exposure and the efficiency of Cre-mediated recombination, we analyzed the expression of AP in RGCs using *R26rtTACreER;Brn3a^CKOAP/+^* and *R26rtTACreER;Brn3b^CKOAP/+^* mice (Figure 2; see Figure 1 of [reference 7] for a schematic of these reporters). Doxycycline was delivered in food pellets at 1.75 mg/g and/or in drinking water at 0.5, 1, or 2 mg/ml. 4HT dissolved in vegetable oil was delivered to pregnant females or to early postnatal mice as a single IP injection. Retina flat mounts were prepared from adult mice, stained histochemically for AP activity, and scored as having high/medium labeling (>50 AP+ RGCs per retina; Figure 2E, F), sparse labeling (1–50 RGCs per retina; Figure 2C), or no labeling (Figure 2B). Various dosing regimens were tested, of which 33 are shown diagrammatically in Figure 2A. For each of the two genotypes tested, the diagrams have been ordered with respect to recombination efficiency, presented in the histograms on the right side of Figure 2A.

From these experiments several patterns emerge. First, initiating doxycycline exposure after approximately embryonic day (E) 5 gives low or no recombination, even if a large dose of 4HT is delivered subsequently [e.g.], [ experiments 1]–[8], [22], [and 23]; (Figure 2B). Second, continuous exposure to doxycycline beginning prior to or at E0 produces recombination in a dose-dependent manner, with some recombination seen even in the absence of 4HT [e.g.], [ experiments 13], [ 18]–[21], [28], [32], [and 33]; (Figure 2F). Third, initiating doxycycline exposure between E2 and E4 and delivering 4HT between E10 and E12 gives dose-dependent recombination that results in a relatively sparse collection of AP+ RGCs suitable for the analysis of single cell morphologies [e.g.], [ experiments 2], [ 3], [ 11], [ 12], [ 24]–[27]; (Figure 2C,E). In particular, in experiments 2, 3, and 11 (with the *Brn3a^CKOAP/+^* target), two experiments showed no labeled neurons and one experiment showed sparse labeling in more than half the retinas. Similarly, in experiments 24, 25, and 27 (with the *Brn3b^CKOAP/+^* target), one third of the retinas have no labeled neurons and two thirds of the retinas have a sparse distribution of labeled neurons.

Interestingly, several retinas with sparse labeling exhibited clusters of AP+ RGCs indicative of early clonal activation or repression (e.g., Figure 2C); an analogous clustering of AP+ neurons and their processes were observed in the brains of several of these mice, as seen in the coronal section through the superior colliculus in Figure 2D. Importantly, clustering of labeled RGCs was observed in retinas that received 4HT after E11, a time when there are already many hundreds of retinal progenitors and lateral migration of cells within a single retinal clone is severely restricted [10]. Thus, the data suggest that the clonal restriction reflects events that occurred earlier in development, such as epigenetic silencing of rtTA expression and/or rtTA-dependent activation of the CreER target in a subset of cells. It seems plausible to suppose that the spatial patterns of clonal restriction might reflect the same epigenetic process that silences CreER expression when doxycycline is not delivered in early gestation. The weak AP staining of all axon bundles in Figures 2B and C arises from a low-level background of AP expression in RGCs from the *Brn3a^CKOAP^* and *Brn3b^CKOAP^* alleles in the absence of Cre-mediated recombination.

In sum, by using an appropriate regimen of doxycycline exposure, the *R26rtTACreER* locus can be used, even with highly recombinogenic target loci, to generate a sparse set of labeled cells at a developmental time point determined by 4HT injection.

### *R26IAP*: A Sensitive Cre-Dependent AP Reporter for a Broad Array of Tissues and Cell Types

To create an AP reporter that is activated by Cre-mediated recombination with an efficiency higher than the relatively inefficient *Z/AP* locus and to eliminate background AP activity in unrecombined cells, a *Rosa26* knock-in allele was constructed with an AP coding region in which the 3′ half is inverted (*R26IAP*; Figure 1C). Upon Cre-mediated recombination, the 3′ half of the coding region, which is flanked by loxP sites in head-to-head orientation, is flipped into the correct orientation in a subset of cells. On the assumption that a variable number of DNA inversion events occurs before dissociation of the Cre/loxP complex, we expect that the recombination process would produce a mixture of correct and inverted orientations in those cells in which recombination has occurred. Therefore, AP expression from the *R26IAP* allele should persist in a subset of recombined cells, with virtually no background AP activity in unrecombined cells. Cre-mediated inversion has been used previously in experiments in which extremely tight control of gene expression was essential, for example in the *in vivo* analysis of oncogenic fusion proteins [12].

**Figure 3:**
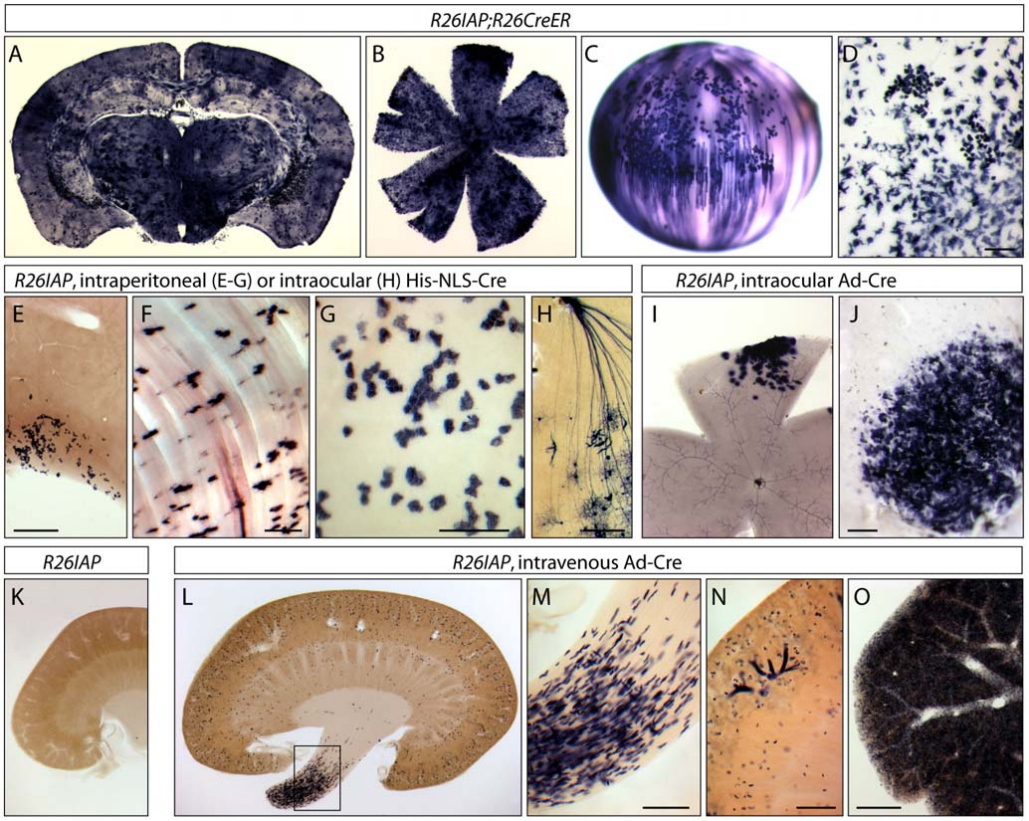
*R26IAP* is a sensitive reporter for Cre-mediated recombination in individual cells. (A–D) *R26CreER;R26IAP* mice either in the absence (A,B) or following intra-ocular injection (C,D) of 4HT show widespread Cre-mediated recombination in diverse cell types. A, brain; B, retina, C, lens, D, cornea. AP+ lens fiber and lens epithelial cells are seen in C. The overall frequency of AP-expressing cells in the brain and retina in the absence of 4HT is ∼0.01%. (E–G) *R26IAP* mice that received an IP injection of ∼184 µg of purified His-NLS-Cre protein show scattered Cre-mediated recombination only in epithelial cells lining the peritoneal cavity or in the muscle fibers of the abdominal wall immediately beneath the peritoneal surface. E, liver; F, abdominal wall; G, stomach. (H) *R26IAP* mice that received an intraocular injection of ∼7 µg of purified His-NLS-Cre protein at ∼P5 show scattered Cre-mediated in RGCs and in occasional astrocytes. RGC axons are converging on the optic nerve head at the top of the panel. (I,J) Retina (I) and cornea (J) following an intra-ocular injection of a replication defective adenovirus expressing Cre. Cre-mediated recombination and AP expression are confined to tissues near the injection site. In the retina, the compact appearance of the AP+ cells and the absence of AP+ axons suggests that most of the labeled cells are astrocytes and/or Muller glia. (K–O) Tissues from *R26IAP* mice in the absence (K) or following IV injection (L–O) of a replication defective adenovirus expressing Cre (Ad-Cre). K–N, kidney; O, liver. The boxed region of the renal pelvis in L is enlarged in M. N shows a region of renal cortex from a different mouse that received an IV injection of Ad-Cre in which Cre-mediated recombination was also observed in a subset of cortical tubules. Scale bars are: 1 mm for E and O; and 200 µm for D,F,G,H,J,M, and N.

As an initial test of the efficiency of Cre-mediated recombination at the *R26IAP* locus, we examined AP expression by histochemical staining of adult tissues in *R26CreER;R26IAP* mice that had not been exposed to 4HT (Figure 3A,B). This analysis revealed 4HT-independent recombination at a frequency of hundreds of cells per retina and ∼1,000 cells per 300 µm vibratome section of brain, roughly similar to the levels observed with *R26CreER;Brn3a^CKOAP/+^* and *R26CreER;Brn3b^CKOAP/+^* mice in the absence of 4HT. As noted in the Introduction, *R26CreER;Z/AP* mice average <1 AP+ cells per retina or 300 µm brain section in the absence of 4HT [10]. In *R26CreER;R26IAP* mice, the number of AP+ cells increased by several orders of magnitude following a single IP injection of 200 µg 4HT at P3, resulting in AP-stained retina flat mounts that are completely opaque (not shown). Recombination in *R26CreER;R26IAP* mice is observed in a wide variety of tissues, as expected for expression of both CreER and AP from the *Rosa26* locus. For example, Figure 3C and D shows scattered AP+ cells in the lens and cornea following intraocular delivery of 6 µg 4HT in adult mice.

### Using *R26IAP* to Assess the Efficiency of Different Methods for Cre Delivery

The relatively high recombination efficiency of the *R26IAP* locus, together with the high sensitivity of AP histochemistry, suggested that the *R26IAP* locus might be generally useful for monitoring the efficiency of Cre-mediated recombination in a variety of experimental contexts. To test this idea, we have used *R26IAP* to compare Cre-mediated recombination following: (a) direct uptake of purified Cre protein fused to a poly-basic peptide (His-NLS-Cre; 13) injected into the peritoneal cavity or into the eye (Figure 3E–H), or (b) infection with a defective adenovirus vector expressing Cre (Ad-Cre) delivered by intravenous or intraocular injection (Figure 3I–O). Each of these methods of Cre administration induced recombination at the *R26IAP* locus, with the cell type, location, and efficiency of recombination reflecting the delivery route and vehicle.

Intraperitoneal (IP) injection of His-NLS-Cre in adulthood led to recombination predominantly in epithelial cells lining the peritoneal cavity, as seen on the surface of the liver, stomach, and abdominal wall (Figure 3E–G); the only AP+ cells not lining the surface epithelium were occasional AP+ abdominal wall muscle fibers (Figure 3F). Similarly, intraocular (i.e. intravitreal) injection of His-NLS-Cre at P5 induced recombination principally in retinal cells in close proximity to the vitreal surface: RGCs, astrocytes, and superficial vascular cells (Figure 3H). Intraocular injection of Ad-Cre in adult mice resulted in transduction of Cre into retinal and corneal cells (Figure 3I and J), and tail vein injection of Ad-Cre in adult mice resulted in efficient recombination in the liver (Figure 3O) and scattered recombination in the kidney, with the highest efficiency in the renal pelvis (Figure 3K–N). Intraocular injections of His-NLS-Cre or Ad-Cre gave highly variable frequencies of recombination within the retina, ranging from few or no recombination events to nearly confluent AP staining across the retina, a variability that most likely reflects variation in needle placement, volume injected, and the fraction of the injected volume retained within the eye. In the absence of His-NLS-Cre protein or Ad-Cre virus or a genetically introduced *Cre* or *CreER* locus, no AP+ cells were observed in any tissues examined from *R26IAP* mice, including brain, liver, stomach, kidney, eye, and abdominal wall.

### The Utility of Brain Atlases Composed of Sparsely Labeled and Genetically Defined Neuronal Subtypes

The role of cholinergic and catecholaminergic (dopaminergic, noradrenergic, and adrenergic) neurotransmission in brain function and disease has been an object of long-standing scientific interest, and, as a result, the anatomy of the cholinergic and catecholaminergic systems has been intensively studied in both normal and diseased brains in a wide variety of species. In most studies, cholinergic and catecholaminergic neurons have been visualized by immunostaining for the biosynthetic enzymes choline acetyl transferase (ChAT) and tyrosine hydroxylase (TH), respectively, a method the reveals the cell bodies and processes of all neurons of the particular neurotransmitter type. Due to the high density and wide coverage of immunoreactive processes, this approach generally reveals only minimal information about the morphologies of individual neurons. In the present study, we have developed a complementary method for visualizing systems of neurons defined by neurotransmitter type. In this method, cell-type specific, but relatively low frequency, Cre-mediated recombination randomly labels a subset of neurons within the population of interest to generate an atlas that reveals representative soma locations, projections, and individual morphologies for all of the neurons of that class.

### Using *ChAT-IRES-CreER;R26IAP* Mice to Produce an Atlas of Cholinergic Neurons

**Figure 4:**
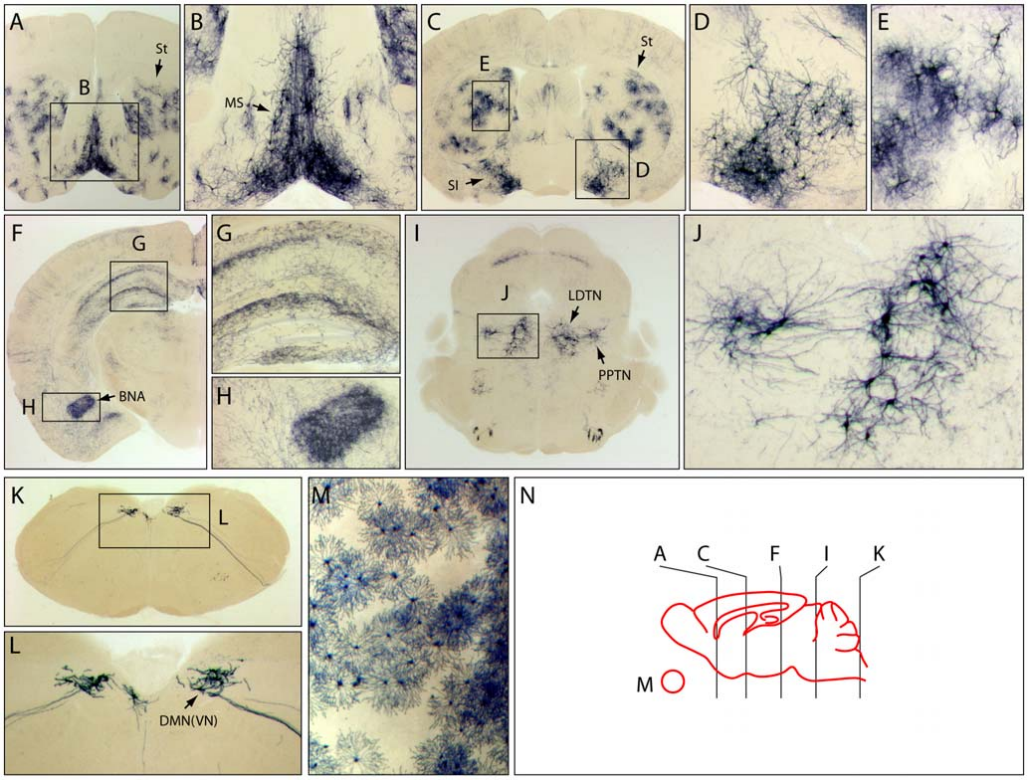
Survey of cholinergic neuronal morphologies and projections in the brain and retina of *R26IAP;ChAT-IRES-CreER* mice. Animals were injected with a single IP injection of 200 µg 4HT at P8. (A,C,F,I,K) 300 µm coronal sections at Bregma positions 1.2, 0, −2.5, −5.0, and −7.8, respectively, as shown schematically in (N). (B,D,E,G,H,J,L) enlargements of boxed regions. (M) In a flat mounted retina, AP-expression is present exclusively in starburst amacrine cells. In the absence of 4HT, retinas were devoid of AP+ cells. BNA, basolateral nucleus of the amygdala; DMN(VN), dorsal motor nucleus of the vagus nerve; LDTN, laterodorsal tegmental nucleus; MS, Medial septum; PPTN; pedunculopontine tegmental nucleus; SI, substantia innominata; St, striatum.

The application of this sampling method to the cholinergic system is illustrated in Figure 4. In this experiment, a *ChAT-IRES-CreER;R26IAP* mouse was injected IP with 200 µg 4HT at P8, and one month later a complete series of 300 µm coronal brain sections was histochemically stained for AP (Figure 4N). The distribution of AP+ cell bodies and processes was found to be in excellent agreement with the distribution of cholinergic neurons and processes observed in mouse and rat brains by ChAT immunostaining [14], [15]. In the following paragraph, we summarize the distribution of the AP+ neurons and processes in the *ChAT-IRES-CreER;R26IAP* brain, proceeding in a rostral to caudal direction.

AP+ neurons with well-defined processes were observed in the medial septum (Figure 4A,B) and in the basal forebrain in and near the substantia innominata (Figure 4C,D). In the striatum, AP+ neurons had both thick tortuous dendrites and a halo of much finer (presumably axonal) processes (Figure 4A,C,E). A large number of fine AP+ processes were found throughout the cortex and hippocampus, as expected for diffuse cholinergic projections originating in the basal forebrain (Figure 4F,G); an especially dense plexus of AP+ processes was present in the basolateral nucleus of the amygdala (Figure 4F-H). Near the pons/midbrain junction, AP+ neurons were localized to the laterodorsal tegmental nucleus and the pedunculopontine tegmental nucleus (Figure 4I,J). In the caudal medulla, AP+ neurons were seen in the dorsal motor nucleus of the tenth (vagus) nerve (Figure 4K,L). In retina flat mounts, AP labeling was confined to starburst amacrine cells, the only cholinergic cell type within the mammalian retina, confirming the specificity of *ChAT-IRES-CreER* expression (Figure 4M).

### Using *TH-IRES-CreER;R26IAP* Mice to Produce an Atlas of Catecholaminergic Neurons

**Figure 5:**
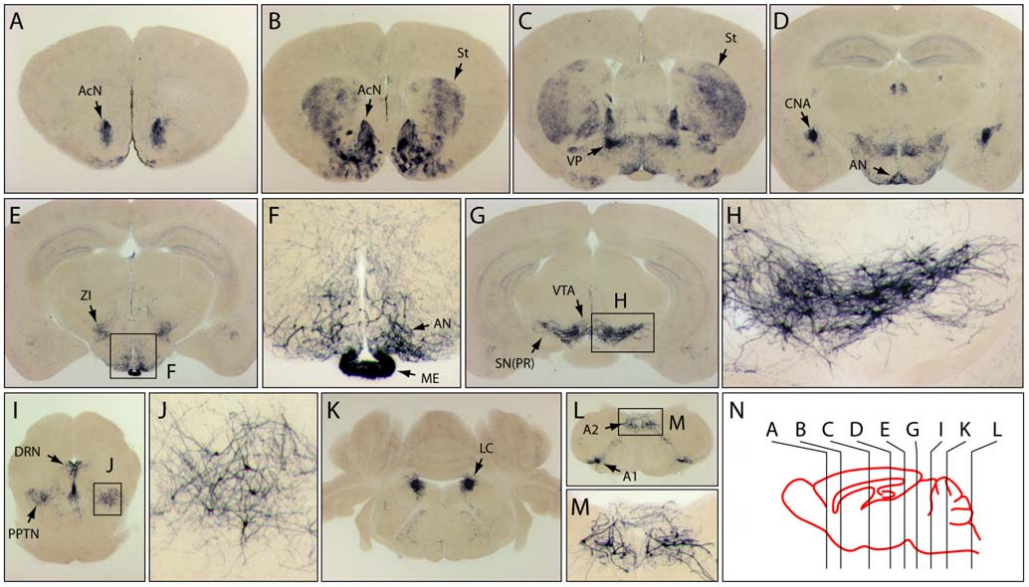
Survey of catecholaminergic neuronal morphologies and projections in the brain of *TH-IRES-CreER;R26IAP* mice. Animals were injected with 100 µg 4HT at P3. (A–E,G,I,K,L) 300 µm coronal sections at Bregma positions 1.7, 1.1, 0, −1.6, −2.2, −3.2, −4.7, −5.5, −8.2, respectively, as shown schematically in (N). (F,H,J,M) enlargements of boxed regions. A1, A1 noradrenergic cell group; A2, A2 noradrenergic cell group; AcN, accumbens nucleus; AN, arcuate nucleus; CNA, central nucleus of the amygdala; DRN, dorsal raphe nucleus; LC, locus coeruleus; ME, median eminence; PPTN, pedunculopontine tegmental nucleus; SN(PR), substantia nigra (pars reticulata); St, striatum; VP, ventral pallidum; VTA, ventral tegmental area; ZI, zona incerta.

To further extend the approach of labeling a subset of neurons within a given neurotransmitter system, we have also generated an atlas of labeled neurons for the catecholaminergic systems by targeting *R26IAP* recombination with *TH-IRES-CreER* following IP injection of 100 µg 4HT at P3. Figure 5 shows a series of 300 µm coronal sections from a *TH-IRES-CreER;R26IAP* brain that was histochemically stained for AP (Figure 5N). The distribution of AP+ cell bodies and processes was found to be in excellent agreement with the distribution of catecholaminergic neurons and processes observed in mouse and rat brains by TH immunostaining [16]–[21]. Below we summarize the distribution of AP+ neurons and processes in the *TH-IRES-CreER;R26IAP* brain, proceeding in a rostral to caudal direction.

In the *TH-IRES-CreER;R26IAP* forebrain, dense AP+ processes, but very few AP+ cell bodies, were seen in the nucleus accumbens, striatum, ventral pallidum, and the central nucleus of the amygdala (Figure 5A–D), as expected for these principal targets of innervation by midbrain dopaminergic and noradrenergic neurons. At the level of the hypothalamus, scattered AP+ cell bodies with a small number of thick processes were present in the arcuate nucleus (Figure 5E and F). Intense AP staining was seen in the median eminence, and AP+ neurons with numerous finer processes were seen in the zona incerta (Figure 5E and F). The greatest number of AP+ neuronal cell bodies were found in a contiguous zone encompassing the substantia nigra pars reticulata and the ventral tegmental area and extending caudally as far as the pedunculopontine tegmental nucleus, nuclei that give rise to the principal dopaminergic projections to the forebrain (Figure 5G–J). At the junction of the midbrain and pons, large numbers of AP+ cells populate the dorsal raphe nucleus, a major source of projections to the nucleus accumbens (Figure 5I). The locus coeruleus, the site with the highest concentration of central noradrenergic neurons, was densely populated with AP+ cells (Figure 5K). Finally, AP+ neurons were observed in the caudal medulla in the locations predicted for A1 and A2 noradrenergic cell groups (Figure 5L and M).

### Using *TH-IRES-CreER;R26IAP* Mice to Visualize the Morphologies of Wide-Field Tyrosine Hydroxylase-Expressing Amacrine Cells

As a further test of the utility of the *R26IAP* line, we have asked whether we could use it to characterize the unusually large arbors of dopaminergic amacrine (DA) cells in the mouse retina. DA cells have been found in the retinas of a wide variety of vertebrates. Both rodent and primate retinas have a distinctive type of TH+ amacrine cells (type 1 cells) characterized by large cell bodies with two groups of neurites: one group forms a simple tree composed of relatively thick dendrites, whereas the second is composed of extremely long, thin, and sparsely branching axon-like processes [22]–[24]. The dendritic and axon-like arbors are confined to the outermost sublamina of the inner plexiform layer. In the primate retina, the dendritic arbors of DA cells have diameters of ∼400 µm near the fovea and ∼600 µm in the periphery, whereas the axon-like arbors encompass a diameter of at least 6 mm, the exact value being difficult to ascertain due to uncertainty in the extent of diffusion of the injected label [24]. Type 1 DA cells are unusually sparse. In the primate retina they have a density of ∼50/mm^2^ near the fovea and about 10/mm^2^ in the periphery, and in the mouse and rabbit they have an average density of ∼20/mm^2^, equivalent to ∼450 and ∼6,000 cells per retina, respectively [24]–[27]. Despite their relatively small numbers, dopamine released by DA cells plays a central role in modulating retinal signaling in response to changes in ambient illumination or circadian cues [28]–[32].

In most mammalian retinas, including the mouse retina, TH immunostaining can been used to identify DA cells, but the extensive intermingling of TH+ processes from different cells precludes the tracing of individual immunolabeled arbors. A similar challenge exists for transgenic approaches in which the TH promoter drives expression of a histochemical or fluorescent reporter [27], [33], [34].

**Figure 6:**
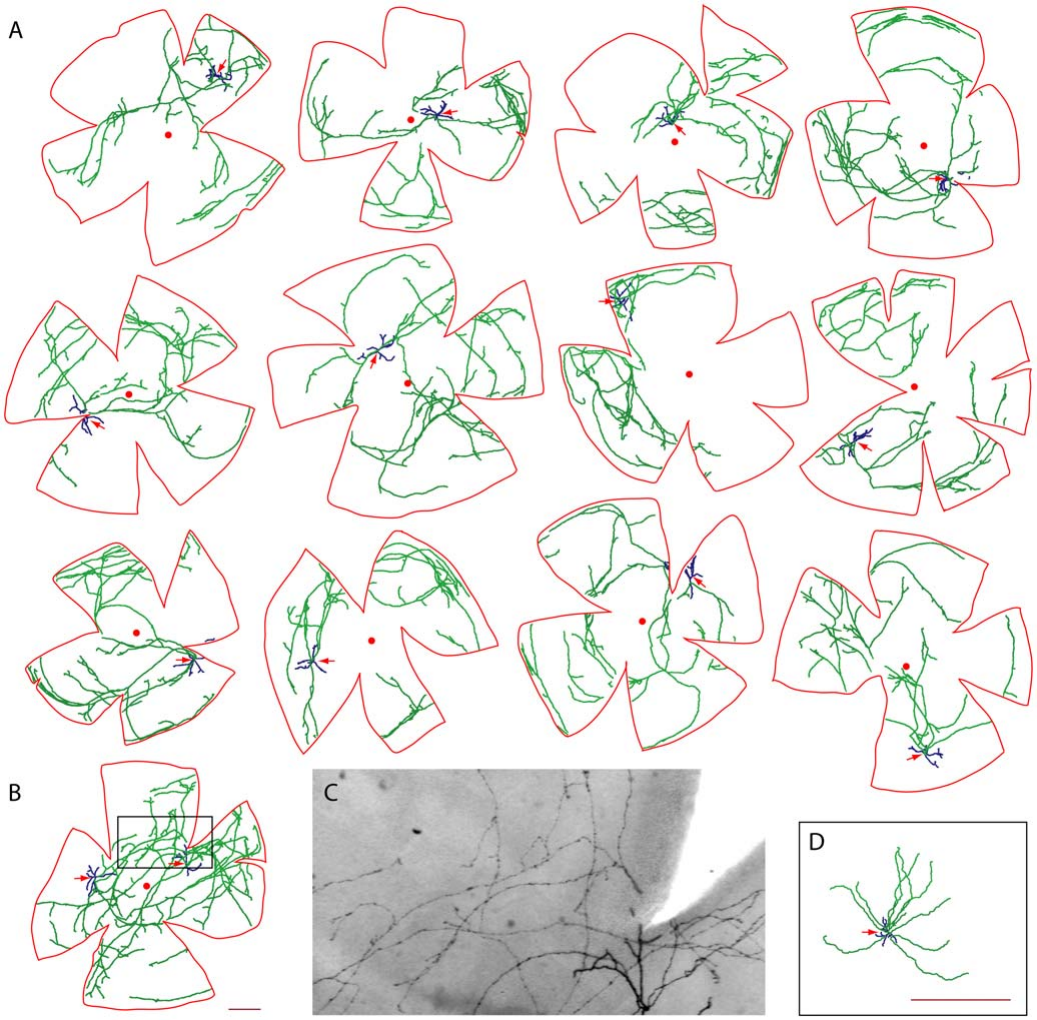
Morphologies of individual AP+ type 1 DA cells from the retinas of *TH-IRES-CreER;R26IAP* mice. A single 100–300 µg 4HT injection was administered IP between P3 and P8; retinas were analyzed ∼6 weeks later. Retina flat mounts were processed for AP histochemistry and clarified in BBBA. Axon-like processes (green) and dendrites (blue) were traced with Neuromantic software. The soma is indicated by a red arrow. The outline of the flattened retina and the location of the optic disc are shown in red. (A) Twelve retinas, each of which had a single type 1 DA cell. (B) A retina with two type 1 DA cells. (C) Image of the boxed region of the retina shown in (B). The dendrites (lower right) are uniformly thicker and more darkly stained than the axon-like processes. (D) A putative type 2 DA cell. Scale bars in B and D, 500 µm. Scale bar in B applies to panels A and B. BBBA causes ∼30% tissue shrinkage.

In the present study, we have used *TH-IRES-CreER;R26IAP* mice injected with 100–300 µg 4HT between P3 and P8 to produce, on average, fewer than one AP+ DA cell per retina. This window of injection times was chosen to roughly match the initial appearance of TH+ amacrine cells in the rodent retina [35]–[37]. Among 120 adult retinas histochemically stained for AP and examined as flat mounts, a total of 147 AP+ cells were observed, and all were amacrine cells. Twenty-five of the AP+ cells exhibited morphologies indicative of type 1 DA cells: a large soma, a relatively simple dendritic arbor, and an extremely large and sparsely branching axon-like arbor, with both arbors confined to the outermost lamina of the inner plexiform layer (Figure 6A–C). This narrow lamination is in contrast to the diverse lamination within the inner plexiform layer that is seen with other large polyaxonal amacrine cells in primate and rodent retinas [5], [38], [39]

The distribution of AP+ type 1 DA cells among the 120 retinas was consistent with a Poisson process: the number of retinas with zero, one, two, or more than two type 1 DA cells was 97, 21, two, and zero, respectively. Fourteen of the type 1 DA cells were traced (Figure 6A and B): 12 from retinas with a single type 1 DA cell, and two from one retina that had a pair of type 1 DA cells. In the retina with two type 1 DA cells (Figure 6B and C), the extensive overlap between the two axon-like arbors precludes an accurate assignment of connectivity for some of the axon-like segments.

**Figure 7:**
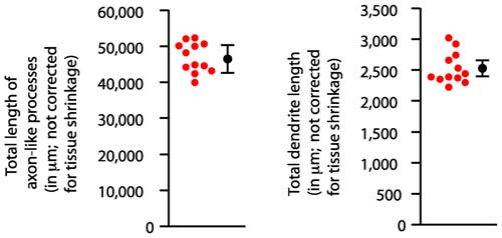
Quantification of axon-like processes and dendrite lengths for type 1 DA cells. Red circles, lengths calculated from the Neuromantic tracing of BBBA clarified retinas. BBBA causes ∼30% tissue shrinkage. Black circle and error bars, mean+/−S.D.

Among the collection of AP+ type 1 DA cells, the relative uniformity of the AP staining intensity along each axon-like process and the sharp termination of AP staining at the presumptive ends of these processes implies that their full extent has been visualized (Figure 6C). In general, the axon-like arbors encompassed regions of the retina both near to and far from the cell body. There were multiple examples of axon-like processes that projected close to the edge of the retina and then continued circumferentially along the retinal margin, suggesting that in the context of a larger retina this axon-like trajectory would have continued radially to greater distances from the cell body (Figure 6A). Despite the apparent absence of a fixed branching pattern, the length distributions for both the dendritic and axon-like arbors were remarkably narrow, with a mean+/−S.D. of 46,908+/−4,175 µm for axon-like processes and 2,532+/−253 for dendrites [Figure 7; these values are uncorrected for the ∼30% tissue shrinkage caused by clearing in benzylbenzoate:benzyl alcohol (BBBA)]. These observations imply the existence of a biochemical control system that precisely calibrates the total length (or mass) of these arbors.

In addition to the 25 type 1 DA cells described above, a variety of other AP+ amacrine cell types were also found in this set of 120 retinas: 110 AII amacrine cells, four starburst amacrine cells, four large field amacrine cells, two “waterfall” amacrine cells, and two narrow field amacrine cells [see ref. 5 for examples of these morphologies]. Among the large field amacrine cells, at least one corresponds to the morphology described for type 2 DA cells (Figure 6D). We ascribe the occasional labeling of non-DA cell types to low level mis-expression of *TH-IRES-CreER* in these other neurons or in neuronal progenitors.

## Discussion

The two new mouse lines described here add to the growing list of genetically engineered mice available for controlled *in vivo* recombination and cell labeling using the Cre-lox system. The *R26rtTACreER* line extends the dynamic range of Cre-mediated recombination efficiencies relative to that of other loci with a constitutively expressed CreER, and the *R26IAP* line complements the widely used Z/AP line by providing substantially higher efficiency Cre-mediated activation of AP. We note that Que and colleagues [40] have recently described the construction of a *Rosa26* knock-in mouse line in which a hPLAP coding region is transcribed from a CMV enhancer/beta-actin promoter after Cre-mediated excision of a loxP-stop-loxP cassette.

It seems likely that for applications requiring low efficiency recombination, the dynamic range and temporal control offered by *R26rtTACreER* represents an improvement over pre-existing lines in which the Tet system controls a constitutively active Cre recombinase [41]–[44]. It will be interesting to extend the *rtTACreER* strategy by targeting this cassette to loci that confer cell-type specific expression. Based on our observations with the *R26rtTACreER* locus, we would predict that this two-stage pharmacologic strategy will also confer tighter control of Cre activity relative to the standard *CreER* cassette at other sites in the genome.

### Factors Affecting the Efficiency of Cre-Mediated Recombination

The roughly one thousand-fold difference between *Z/AP* and *R26IAP* recombination frequencies emphasizes the large effect of chromosomal location and/or target gene sequence on the efficiency of Cre-mediated recombination. This effect is substantially larger than the variation typically observed among target loci [45]. A second variable that affects the efficiency of Cre-mediated recombination is developmental age. With multiple target loci for which the efficiency of Cre-medated recombination can be easily quantified with AP histochemistry - including *Brn3a*, *Brn3b*, *Z/AP*, and *Fz5* - we have consistently observed a decline in efficiency with increasing postnatal age when tested with a variety of *CreER* lines, including *R26CreER*, *NFL-IRES-CreER*, and *ChAT-IRES-CreER* (our unpublished observations). As the expression of the *Rosa26*, *NFL*, and *ChAT* genes persists in adulthood, the observed decline in recombination efficiency with age is unlikely to arise from the loss of CreER expression. With the *R26rtTACreER* system we observe an age dependent silencing that can be inhibited by initiating doxycycline exposure – and presumably rtTA activated transcription – in early embryonic development.

These age-dependent phenomena likely represent progressive epigenetic changes in gene structure. A recent comparison of recombination efficiencies among the *Z/AP*, *Z/EG*, and *R26R-EYFP* loci in hematopoietic cells found a similar decline in recombination efficiency with age, as well as a strong correlation between lower recombination efficiency in adulthood and increased cytosine methylation, suggesting that age-dependent changes in chromatin structure can render loxP targets less accessible to Cre recombinase [46]. Consistent with our experience with the *R26rtTACreER* locus, studies of rtTA activation of target genes reveals a progressive silencing in neurons that can be overcome either by maintaining a basal level of target gene expression throughout development or by providing very high levels of rtTA [47].

In studies in which highly efficient Cre-mediated recombination is desirable, the use of a constitutively active Cre-recombinase - even in combination with an inefficient loxP target such as *Z/AP* - usually suffices to produce complete or nearly complete recombination [8]. When temporal control of recombination is also required and the CreER system is used, obtaining highly efficient recombination is more difficult and can require near-toxic doses of 4HT administered over multiple days [3].

By contrast, for sparse cell labeling experiments, the challenge is reversed: the problem is now to reduce the efficiency of 4HT-dependent Cre-mediated recombination and eliminate any background of 4HT-independent recombination. As shown in Figure 3A and B, when using *CreER* alleles with relatively high levels of expression (e.g. *R26CreER*) in combination with relatively efficient loxP targets (e.g. *R26IAP*), background recombination is substantial even in the absence of 4HT. Using a less efficient loxP target, such as *Z/AP*, reduces the recombination level so that there are few or no labeled cells in the absence of 4HT [10]. However, in our experience, the *Z/AP* locus is unusual in this respect, and most loxP target loci more closely resemble *R26IAP* in recombination efficiency. Long and Rossi [46] have suggested that the unusually inefficient recombination efficiencies of the *Z/AP* and *Z/EG* loci reflect the high CpG content of the *E. coli lacZ* coding region, which is present as part of the beta-geo coding region in both loci and may promote somatic cytosine methylation and epigenetic silencing.

A second strategy for reducing recombination efficiency focuses on reducing CreER expression, either by using an *IRES-CreER* knock-in allele, which likely decreases translation inefficiency, or, as in the R26rtTACreER locus, by overlaying a second level of transcriptional regulation. Both of these approaches for reducing CreER can eliminate background recombination in the absence of 4HT. Finally, combining both an inefficient loxP target (*Z/AP*) and low CreER expression reduces recombination to extremely low levels, such that fewer than one hundred neurons can be reproducibly labeled per brain depending on the *IRES-CreER* knock-in and the time and dose of 4HT [4].

### Sparse Labeling of Defined Neuronal Types as a Tool to Survey Changes in CNS Structure

A method for visualizing the morphologies of a large and representative sampling of neurons of a genetically defined type has the potential to fill a hitherto vacant niche in neuro-anatomic analyses of brain structure. Historically, the analysis of neuronal morphology has been performed either by sparse labeling methods that are not cell type specific (e.g. the Golgi stain) or by filling individual neurons with a tracer using a whole cell patch electrode, a method that is extremely laborious and not easily adaptable as a survey tool [e.g. ref. 48]. These classic methods also suffer from the problem of incomplete filling of large and/or complex neuronal processes. At the other end of the spectrum with respect to the number of labeled cells, immunostaining for markers such as ChAT or TH reveals all of the cells of a given type, but the high density of labeled cells and their interdigitating processes generally preclude an assessment of individual morphologies. Moreover, the immunostaining approach is limited to those antigens that are relatively abundant, cytosolic, and cell-type specific. Thus, many molecular markers that distinguish neuronal types, such as transcription factors, or various low abundance or transiently expressed proteins cannot be used directly to reveal the morphologies of the cells in which they are [or were] expressed. While this last challenge is being partially addressed by the creation of BAC transgenic lines that express GFP or Cre in defined subsets of cells [e.g. refs 49], [50], the challenge of visualizing individual neuronal morphologies can only be met by a method that generates a relatively sparse collection of labeled neurons.

The strategy of generating a sparsely labeled sampling of a single neuronal type – what we refer to here as an atlas of morphologies - is illustrated here for cholinergic and catecholaminergic neurons. The method takes advantage of the flexibility afforded by 4HT dosing to noninvasively generate a collection of several dozen to several hundred labeled neurons throughout the entire CNS. Current evidence suggests that the 1–2 month delay between Cre-mediated recombination and histologic analysis provides sufficient time for AP to uniformly populate the plasma membrane of all dendritic and axonal processes even among the largest neurons [4]. This approach could be used to survey the survival and morphology of defined classes of neurons in mouse models of a variety of CNS diseases or insults, including inherited disease, stroke, infection, drug toxicity, and sensory deprivation. For unilateral perturbations - such as a surgical lesion, localized drug injection, or recombinant virus injection – labeled cells ipsilateral and contralateral to the site of the manipulation can be compared.

### The Morphologies of Type 1 DA Cells in the Mouse Retina

DA cells, first defined as dopamine-accumulating neurons and subsequently shown to express TH, have been extensively studied in a variety of species [22]–[25], [51]. Their wide arbors and diffuse coverage likely reflect the role of dopamine as a neuromodulator that acts on a broad and still incompletely enumerated group of target cells to modulate retinal sensitivity. The best-characterized effect of dopamine in the retina is in mediating the light-dependent uncoupling of horizontal and amacrine cell gap junctions [reviewed in ref. 28]. Recent work suggests that retinal dopamine release is regulated by both ambient light level and by the circadian clock [29]–[32].

To the best of our knowledge, the full morphology of individual type 1 DA cells has not been previously reported in any species owing to uncertainties regarding the completeness of tracer filling. Dacey has estimated the diameter of the type 1 primate DA cell axon-like arbor at ∼8–10 mm based on a quantitative analysis of arbor density, cell density, and the morphology of HRP filled cells [24]. The cell morphologies determined here indicate that the typical type 1 DA cell in the mouse retina has axon-like processes that extend both radially and circumferentially, forming a sparse arbor that generally encompasses a substantial fraction of the retinal area and has a total length of ∼6.5 cm in a retina with a flat mounted diameter of ∼5 mm (Figure 7; values corrected for tissue shrinkage in BBA). If the arbors were artificially straightened and directed radially, they would extend to a distance of at least several millimeters from the cell body (Figure 6). On the assumption that signals received by the dendrites of type 1 DA cells are transmitted along the full length of their axon-like arbors, then it would appear that activation of even a small number of these cells would produce a signal with a relatively uniformly spatial output across the retina, a property consistent with the known neuromodulatory functions of retinal dopamine.

In relation to the cell biological mechanisms that control the development of neuronal morphology, the large and irregularly shaped axonal arbors of the type 1 DA cells would appear to rule out any constraints on axonal trajectories based on homotypic signaling, as observed for axonal and dendritic arbors in Drosophila [52], [53] and as postulated for a variety of cell types in the mammalian retina [54]. However, an interesting observation regarding type 1 DA cells is the very narrow length distributions of the dendrites and axon-like processes (Figure 7). These distributions suggest that there is a stringent control mechanism that regulates the total length of each class of neuritic process, even among cell types in which the branching pattern and coverage geometry is highly variable. The hypothesized control mechanism could, for example, reflect a genetic program that precisely controls the production of one or more molecules that are limiting for process formation.

## Methods

### ES Cells and Mouse Husbandry

Knock-in constructs were designed with inserts at the *Xba* I site at the *Rosa26* locus as indicated in Figure 1A. ES cells were electroporated and colonies were selected in G418 and screened by Southern blot hybridization with the 5′ flanking probe shown in Figure 1A. Karyotypically normal ES cells were used for blastocyst injection. Following germline transmission of the targeted allele, the *PGK*-*neo* cassette of *R26rtTACreER* was excised by crossing to mice expressing Flp in the germline. The *R26rtTACreER* and *R26IAP* lines have been maintained as homozygous stocks on a mixed Sv129×C57BL/6 background. Both lines have been deposited at the Jackson Laboratories and are freely available. Mice were handled and housed in accordance with the Johns Hopkins University Animal Care and Use Protocols and IACUC guidelines.

### Genotyping

PCR primers for genotyping are as follows. *R26IAP* [sense strand, located in the first exon of AP: GGCAACGAGGTCATCTCCGTGATGAA; antisense strand, located in the intron in the inverted configuration: GGTTGCCTGGGTCTGAGCTAGTGCC; product size: 290 bp); *R26rtTATetCreER* [sense strand: CCCTCGTGATCTGCAACTCCAGTC ( = TB29); antisense strand: TAGAATCGGTGGTAGGTGTC; product size: 700 bp]; WT [(i.e. the untargeted) Rosa26 locus [sense strand: TB29, listed above; antisense strand: GGAGCGGGAGAAATGGATATG; product size: 500 bp]. PCR was performed with Advantage 2 Polymerase (Clontech, La Jolla, CA) using 35 cycles of 30 seconds denaturation at 94°C, 30 second annealing at 60°C, and 1 minute elongation at 72°C.

### His-NLS-Cre and Ad-Cre

An *E. coli* expression plasmid encoding His-NLS-Cre [13] was a kind gift of Dr. H. Earl Ruley (Vanderbilt University). His-NLS-Cre expressed in E coli was purified to >90% homogeneity by nickel chelate affinity chromatography. The protein was eluted in 500 mM imidazole, and concentrated and desalted by ultrafiltration against PBS supplemented with 150 mM NaCl. For IP injection of His-NLS-Cre, 100 µl of a 1.84 µg/µl stock was injected per adult mouse; for intraocular injection 2 µl of a 3.52 µg/µl stock was injected per eye at P5 using a 30 gauge needle. For IV injection into the tail vein, 400 µl of an Ad-Cre stock (3×10^9^ plaque forming units/ml; Virenz, Baltimore, MD) was injected per adult mouse; for intraocular injection, 1.5 µl of the same virus stock was injected per eye at >P21 with a glass micro-needle. Tissues were analyzed >14 days later for IP His-NLS-Cre and IV Ad-Cre injections, and >10 days later for intraocular injections.

### Doxycycline and 4HT Delivery

Doxycycline (Sigma, St. Louis, MO) was mixed with drinking water and/or delivered as premixed food pellets (Research Diets, Inc., New Brunswick, NJ). For experiments with *R26rtTACreER* mice, 4HT (98% pure) was dissolved in ethanol, mixed with sunflower seed oil (Sigma, St. Louis, MO), centrifuged under vacuum to remove the ethanol, and delivered as a single IP injection. For experiments with *R26IAP* mice, the same protocol was followed except that 70% purity 4HT (Sigma) was used for some experiments. For *R26IAP* mice, IP injections of 4HT were performed between P3 and P8 at a dose of 0.03–0.04 mg/g body weight.

### Tissue Processing

Tissue processing and AP staining were performed essentially as described in ref. 10. For tissues other than eyes, mice were anesthetized and subjected to transcardiac perfusion with 4% paraformaldehyde in PBS. The brain, liver, or kidney was set in a block of 3% low-melting point agarose in PBS and a series of 300 µm sections were cut with a vibratome. Segments of abdominal wall and stomach were processed intact. Retinas were dissected from eyes that had been immersion fixed in 4% paraformaldehyde in PBS, and were gently flattened with a plastic mesh. Tissues in PBS were heated in a water bath at 69°C for 90 minutes to inactivate endogenous AP and, for those embedded in low melting point agarose, to melt the agarose surrounding the tissue; heat-inactivated tissues were then reacted in NBT/BCIP for several hours to overnight at room temperature with gentle agitation. After washing and post-fixation, tissues were dehydrated with a graded ethanol series, and clarified in 2∶1 benzylbenzoate:benzyl alcohol (BBBA). For long-term storage, samples were returned to ethanol.

### Microscopy and Neurite Tracing

Bright-field images were captured on a Zeiss dissecting microscope equipped with OpenLab software, and bright-field and DIC images were captured on a Zeiss Axio Imager Z1 microscope equipped with a motorized stage and AxioVision software. For retina flat mounts, a montage of 5X images was obtained with the Zeiss Axio Imager Z1 microscope using an X-Y stage and the mosaics module and assembled using AxioVision software. For type 1 DA cells, in which dendrites and axon-like processes are confined to a single sublamina of the IPL, the AP-labeled neurites were traced in the X–Y plane without loss of information using Neuromantic software (www.rdg.ac.uk/neuromantic/). For coronal brain sections, Bregma positions and neuroanatomic structures were assigned as indicated in ref. 15.

## References

[1] Branda CS, Dymecki SM (2004). Talking about a revolution: the impact of site-specific recombinases on genetic analysis in mice.. Dev Cell.

[2] Feil R, Brocard J, Mascrez B, LeMeur M, Metzger D (1996). Ligand-activated site-specific recombination in mice.. Proc Natl Acad Sci USA.

[3] Hayashi S, McMahon AP (2002). Efficient recombination in diverse tissues by a tamoxifen-inducible form of Cre: a tool for temporally regulated gene activation/inactivation in the mouse.. Dev Biol.

[4] Rotolo T, Smallwood PM, Williams J, Nathans J (2008). Genetically-directed, cell type-specific sparse labeling for the analysis of neuronal morphology.. PLoS One.

[5] Badea T, Nathans J (2004). Quantitative analysis of neuronal morphologies in the mouse retina visualized using a genetically directed reporter.. J Comp Neurol.

[6] Liu C, Wang Y, Smallwood PM, Nathans J (2008). An essential role for Frizzled5 in neuronal survival in the parafascicular nucleus of the thalamus.. J Neurosci.

[7] Badea TC, Cahill H, Ecker J, Hattar S, Nathans J (2009). Distinct roles of transcription factors brn3a and brn3b in controlling the development, morphology, and function of retinal ganglion cells.. Neuron.

[8] Lobe CG, Koop KE, Kreppner W, Lomeli H, Gertsenstein M (1999). Z/AP, a double reporter for cre-mediated recombination.. Dev Biol.

[9] Friedrich G, Soriano P (1991). Promoter traps in embryonic stem cells: a genetic screen to identify and mutate developmental genes in mice.. Genes Dev.

[10] Badea T, Wang Y, Nathans J (2003). A noninvasive genetic/pharmacologic strategy for visualizing cell morphology and clonal relationships in the mouse.. J Neurosci.

[11] Baron U, Bujard H (2000). Tet repressor-based system for regulated gene expression in eukaryotic cells.. Methods Enzymol.

[12] Forster A, Pannell R, Drynan LF, Codrington R, Daser A (2005). The invertor knock-in conditional chromosomal translocation mimic.. Nat Methods.

[13] Lin Q, Jo D, Gebre-Amlak KD, Ruley HE (2004). Enhanced cell-permeant Cre protein for site-specific recombination in cultured cells.. BMC Biotechnol.

[14] Butcher LL (1995). Cholinergic neurons and networks..

[15] Paxinos G, Franklin KBJ (2001). The Mouse Brain in Stereotaxic Coordinates, second ed..

[16] Hökfelt T, Johansson O, Fuxe K, Goldstein M, Park D (1976). Immunohistochemical studies on the localization and distribution of monoamine neuron systems in the rat brain. I. Tyrosine hydroxylase in the mes- and diencephalon.. Med Biol.

[17] Hökfelt T, Johansson O, Fuxe K, Goldstein M, Park D (1977). Immunohistochemical studies on the localization and distribution of monoamine neuron systems in the rat brain II. Tyrosine hydroxylase in the telencephalon.. Med Biol.

[18] Trulson ME, Cannon MS, Raese JD (1985). Identification of dopamine-containing cell bodies in the dorsal and median raphe nuclei of the rat brain using tyrosine hydroxylase immunochemistry.. Brain Res Bull.

[19] Stratford TR, Wirtshafter D (1990). Ascending dopaminergic projections from the dorsal raphe nucleus in the rat.. Brain Res.

[20] Heimer L, Zahm DS, Alheid GF (1995). Basal ganglia..

[21] Oldfield BJ, McKinley MJ (1995). Circumventricular organs..

[22] Versaux-Botteri C, Martin-Martinelli E, Nguyen-Legros J, Geffard M, Vigny A (1986). Regional specialization of the rat retina: catecholamine-containing amacrine cell characterization and distribution.. J Comp Neurol.

[23] Mariani AP, Hokoc JN (1988). Two types of tyrosine hydroxylase-immunoreactive amacrine cell in the rhesus monkey retina.. J Comp Neurol.

[24] Dacey DM (1990). The dopaminergic amacrine cell.. J Comp Neurol.

[25] Tauchi M, Madigan NK, Masland RH (1990). Shapes and distributions of the catecholamine-accumulating neurons in the rabbit retina.. J Comp Neurol.

[26] Casini G, Brecha NC (1992). Postnatal development of tyrosine hydroxylase immunoreactive amacrine cells in the rabbit retina: II. Quantitative analysis.. J Comp Neurol.

[27] Gustincich S, Feigenspan A, Wu DK, Koopman LJ, Raviola E (1997). Control of dopaminergic release in the retina: a transgenic approach to neural networks.. Neuron.

[28] Baldridge WH, Vaney DI, Weiler R (1998). Thee modulation of intracellular coupling in the retina.. Seminars Cell Dev Biol.

[29] Storch KF, Paz C, Signorovitch J, Raviola E, Pawlyk B (2007). Intrinsic circadian clock of the mammalian retina: importance for retinal processing of visual information.. Cell.

[30] Zhang DQ, Zhou TR, McMahon DG (2007). Functional heterogeneity of retinal dopaminergic neurons underlying their multiple roles in vision.. J Neurosci.

[31] Zhang DQ, Wong KY, Sollars PJ, Berson DM, Pickard GE (2008). Intraretinal signaling by ganglion cell photoreceptors to dopaminergic amacrine neurons.. Proc Natl Acad Sci USA.

[32] Cameron MA, Pozdeyev N, Vugler AA, Cooper H, Iuvone PM (2009). Light regulation of retinal dopamine that is independent of melanopsin phototransduction.. Eur J Neurosci.

[33] Gelman DM, Noaín D, Avale ME, Otero V, Low MJ (2003). Transgenic mice engineered to target Cre/loxP-mediated DNA recombination into catecholaminergic neurons.. Genesis.

[34] Zhang DQ, Stone JF, Zhou T, Ohta H, McMahon DG (2004). Characterization of genetically labeled catecholamine neurons in the mouse retina.. Neuroreport.

[35] Foster GA, Schultzberg M, Goldstein M, Hökfelt T (1985). Differential ontogeny of three putative catecholamine cell types in the postnatal rat retina.. Brain Res.

[36] Mitrofanis J, Maslim J, Stone J (1988). Catecholaminergic and cholinergic neurons in the developing retina of the rat.. J Comp Neurol.

[37] Wulle I, Schnitzer J (1989). Distribution and morphology of tyrosine hydroxylase-immunoreactive neurons in the developing mouse retina.. Brain Res Dev Brain Res.

[38] Dacey DM (1989). Axon-bearing amacrine cells of the macaque monkey retina.. J Comp Neurol.

[39] Lin B, Masland RH (2006). Populations of wide-field amacrine cells in the mouse retina.. J Comp Neurol.

[40] Que J, Wilm B, Hasegawa H, Wang F, Bader D (2008). Mesothelium contributes to vascular smooth muscle and mesenchyme during lung development.. Proc Natl Acad Sci USA.

[41] Schönig K, Schwenk F, Rajewsky K, Bujard H (2002). Stringent doxycycline dependent control of CRE recombinase in vivo.. Nucleic Acids Res.

[42] Bäckman CM, Zhang Y, Malik N, Shan L, Hoffer BJ (2009). Generalized tetracycline induced Cre recombinase expression through the ROSA26 locus of recombinant mice.. J Neurosci Methods.

[43] Lin C, Yin Y, Chen H, Fisher AV, Chen F (2009). Construction and characterization of a doxycycline-inducible transgenic system in Msx2 expressing cells.. Genesis.

[44] Rao P, Monks DA (2009). A tetracycline-inducible and skeletal muscle-specific Cre recombinase transgenic mouse.. Dev Neurobiol.

[45] Vooijs M, Jonkers J, Berns A (2001). A highly efficient ligand-regulated Cre recombinase mouse line shows that LoxP recombination is position dependent.. EMBO Reports.

[46] Long MA, Rossi FM (2009). Silencing inhibits Cre-mediated recombination of the Z/AP and Z/EG reporters in adult cells.. PLoS One.

[47] Zhu P, Aller MI, Baron U, Cambridge S, Bausen M (2007). Silencing and un-silencing of tetracycline-controlled genes in neurons.. PLoS One.

[48] MacNeil MA, Heussy JK, Dacheux RF, Raviola E, Masland RH (1999). The shapes and numbers of amacrine cells: matching of photofilled with Golgi-stained cells in the rabbit retina and comparison with other mammalian species.. J Comp Neurol.

[49] Gong S, Zheng C, Doughty ML, Losos K, Didkovsky N (2003). A gene expression atlas of the central nervous system based on bacterial artificial chromosomes.. Nature.

[50] Gong S, Doughty M, Harbaugh CR, Cummins A, Hatten ME (2007). Targeting Cre recombinase to specific neuron populations with bacterial artificial chromosome constructs.. J Neurosci.

[51] Kolb H, Cuenca N, Wang HH, Dekorver L (1990). The synaptic organization of the dopaminergic amacrine cell in the cat retina.. J Neurocytol.

[52] Matthews BJ, Kim ME, Flanagan JJ, Hattori D, Clemens JC (2007). Dendrite self-avoidance is controlled by Dscam.. Cell.

[53] Millard SS, Flanagan JJ, Pappu KS, Wu W, Zipursky SL (2007). Dscam2 mediates axonal tiling in the Drosophila visual system.. Nature.

[54] Poché RA, Reese BE (2009). Retinal horizontal cells: challenging paradigms of neural development and cancer biology.. Development.

